# Control of Dynamic Limb Motion Using Fatigue-Resistant Asynchronous Intrafascicular Multi-Electrode Stimulation

**DOI:** 10.3389/fnins.2016.00414

**Published:** 2016-09-13

**Authors:** Mitchell A. Frankel, V John Mathews, Gregory A. Clark, Richard A. Normann, Sanford G. Meek

**Affiliations:** ^1^Department of Mechanical Engineering, University of UtahSalt Lake City, UT, USA; ^2^School of Electrical Engineering and Computer Science, Oregon State UniversityCorvallis, OR, USA; ^3^Department of Bioengineering, University of UtahSalt Lake City, UT, USA

**Keywords:** neuroprosthesis, closed-loop control, peripheral nerve, asynchronous stimulation, intrafascicular stimulation

## Abstract

Asynchronous intrafascicular multi-electrode stimulation (aIFMS) of small independent populations of peripheral nerve motor axons can evoke selective, fatigue-resistant muscle forces. We previously developed a real-time proportional closed-loop control method for aIFMS generation of isometric muscle force and the present work extends and adapts this closed-loop controller to the more demanding task of dynamically controlling joint position in the presence of opposing joint torque. A proportional-integral-velocity controller, with integrator anti-windup strategies, was experimentally validated as a means to evoke motion about the hind-limb ankle joint of an anesthetized feline via aIFMS stimulation of fast-twitch plantar-flexor muscles. The controller was successful in evoking steps in joint position with 2.4% overshoot, 2.3-s rise time, 4.5-s settling time, and near-zero steady-state error. Controlled step responses were consistent across changes in step size, stable against external disturbances, and reliable over time. The controller was able to evoke smooth eccentric motion at joint velocities up to 8 deg./s, as well as sinusoidal trajectories with frequencies up to 0.1 Hz, with time delays less than 1.5 s. These experiments provide important insights toward creating a robust closed-loop aIFMS controller that can evoke precise fatigue-resistant motion in paralyzed individuals, despite the complexities introduced by aIFMS.

## Introduction

Paralysis due to spinal cord injury or stroke can leave a person with intact peripheral nerves and muscles, but deficient volitional motor control, thereby reducing their health and quality of life. Many paralyzed individuals consider restoration of lost basic motor functions like grasping and walking as important behaviors that could improve their quality of life (Anderson, 2004). Functional neuromuscular stimulation (FNS) has been extensively investigated as a means to aid and restore lost motor function to paralyzed individuals (Prodanov et al., 2003; Navarro et al., 2005). FNS devices have provided benefit to paralyzed individuals (Popovic et al., 2001), though their use has been limited by rapid muscle fatigue due to high stimulation frequencies and inverse recruitment of fast-fatiguing fibers first, as well as and poorly evoked movement kinematics (Mortimer, 1981; Bhadra and Peckham, 1997; Brissot et al., 2000). Most of the limitations can be attributed to the surface or intramuscular stimulating electrodes used, which achieve poor muscle selectivity and a low ability to grade muscle force output, making current clinical FNS-based systems behave like on/off stimulators (Bhadra and Peckham, 1997). Other FNS stimulation methods, such as intraspinal microstimulation, have been shown to be effective for fatigue-resistant feline walking by evoking synergistic motions, but are unable to control individual muscle or joint activity, which is necessary for dexterous control of movement (Mazurek et al., 2012).

Advances in high-electrode-count peripheral neural interfaces, such as the Utah Slanted Electrode Array (USEA) used in this study, have enabled the selective activation of small groups of motor-units within a single targeted muscle (Branner et al., 2001; Dowden et al., 2009). Smoothly graded muscle forces can be generated by modulating the stimulus intensity delivered via a subset of selected implanted electrodes, which proportionally activate groups of motor units within the targeted muscle. Relatively high-frequency electrical stimulation is required to evoke smooth tetanic muscle forces when the stimulation is delivered via a surface, epimysial, or extraneural electrode, or via a single stimulating intraneural electrode (Cooper and Eccles, 1930; Rack and Westbury, 1969; Brown et al., 1999), and this contributes to rapid muscle fatigue. However, smooth fatigue-resistant muscle forces can be evoked by asynchronously activating multiple small populations of motor-unit groups within a single muscle by stimulating via multiple intrafascicularly implanted microelectrodes at a lower stimulation amplitude and low per-electrode frequency, with a high overall composite stimulation frequency (Yoshida and Horch, 1993; Brown et al., 1999; Wise et al., 2001; McDonnall et al., 2004). This method of asynchronous intrafascicular multi-electrode stimulation (aIFMS) provides a more biomimetic form of muscle activation than does the stimulation strategies used in current clinical FNS-based neuroprostheses. Although it may be more difficult for aIFMS to evoke maximal-forces when compared with traditional methods where motor-units are activated simultaneously, comparison studies have demonstrated that asynchronous stimulation can evoke physiologically-relevant forces near or at maximal levels while providing long-lasting fatigue resistance (Lind and Petrofsky, 1978; McDonnall et al., 2004). More recently, aIFMS has been successfully used to evoke fatigue-resistant, physiologically-relevant muscle forces using USEAs in acute, and chronic studies (Frankel et al., 2011; Normann et al., 2012).

Before an aIFMS system can become clinically viable for evoking coordinated movements, one must be able to control the stimulation parameters delivered via multiple selected electrodes. The dynamic muscle response to single electrode stimulation has been well studied, modeled, and used to create control algorithms (Rack and Westbury, 1969; Zajac, 1989; Bobet et al., 1993; Riener et al., 1996; Yoshida and Horch, 1996; Ferrarin and Pedotti, 2000; Schauer et al., 2005). Unfortunately, these models do not extend well to multi-electrode stimulation because of currently unmodeled dynamics, such as axonal activation overlap between stimulating electrodes and the non-linear combination of muscle forces evoked by the multiple stimulating electrodes (Parmiggiani and Stein, 1981). FNS-based neuroprostheses also contain poorly modeled, non-linear, time-varying processes such as potentiation and fatigue (Gordon et al., 1990; Giat et al., 1993). Because of these issues, *a priori* determination of aIFMS parameters to evoke precise muscle forces or limb motion is currently not possible; hence, closed-loop control methods are required.

Recently, we developed a multiple-input, single-output (MISO) real-time closed-loop control method for determining aIFMS per-electrode stimulation intensities (stimulus pulse durations) to evoke precise, fatigue-resistant, isometric muscle forces in an anesthetized feline (Frankel et al., 2011), a commonly used model of human paralysis. Because this work was successful in evoking isometric forces, it presented a foundation for extending the aIFMS control strategy to the necessary non-isometric muscle forces required for many real-world movements that involve dynamic limb motion, which present additional and difficult control challenges due to the non-linear force-length-velocity profile of skeletal muscle (Veltink et al., 1992; Durfee and Palmer, 1994; Chang et al., 1997).

In this study, we extend the force-feedback proportional aIFMS control strategy to a proportional plus integral plus velocity controller (PIV) for dynamic joint-angle feedback control that includes integrator anti-windup strategies to improve system response characteristics. PID controllers have been used to control FNS for dynamic upper-limb and lower-limb movements using single-electrode per muscle strategies, even in the presence of fatigue and system non-linearities (Veltink, 1991; Watanabe et al., 2005; Schiaffino and Tabernig, 2013), though these have not been extended to multi-electrode per muscle studies. By using the velocity of the evoked joint-angle trajectory to create damping, as opposed to the derivative of the error used in PID controllers, our PIV controller avoids a large error derivative that occurs when there are sharp discontinuities in desired position, such as for step trajectories (Nise, 2004). To evaluate the controller used in this study, we selected desired trajectories that are physiologically relevant to normal human lower-limb movements, such as steps, which are relevant to the ability to hold a stance; ramps, which are relevant for controlled sit-to-stand and stand-to-sit (eccentric) motions; and periodic trajectories, which are relevant to gait movements.

## Methods

### Surgical preparation and electrode array implantation

Chronic survival experiments were conducted on an adult female feline using procedures approved by the University of Utah Institutional Animal Care and Use Committee. A 100-electrode USEA (Normann et al., 2005), was chronically implanted in the left sciatic nerve (Rousche and Normann, 1992; Branner et al., 2004). The feline used in this study was also used in other chronic USEA research, and the experiments performed in this study were conducted 3.5 and 4 months post-implantation (19 days apart). The feline was recovered from anesthesia after implantation and after the two experimental sessions, and provided routine feeding and exercise to keep weight and muscle tone at healthy states.

The animal was anesthetically induced with an intramuscular injection of Telazol (Fort Dodge Animal Health, Fort Dodge, USA) at a dosage of 10 mg/kg. The animal was then intubated and mechanically ventilated. Anesthesia was maintained with Isoflorane (Hospira, Lake Forest, USA) at a level of 0.5–1.5%, which has been shown to suppress spinal reflexes (Zhou et al., 1997). Fluid and blood sugar levels were maintained via an intravenous drip of lactated Ringer's solution at a rate of 10 ml/kg/hr. Vital signs were monitored and recorded every 15 min to assess the depth of anesthesia and animal status.

**Figure 1:**
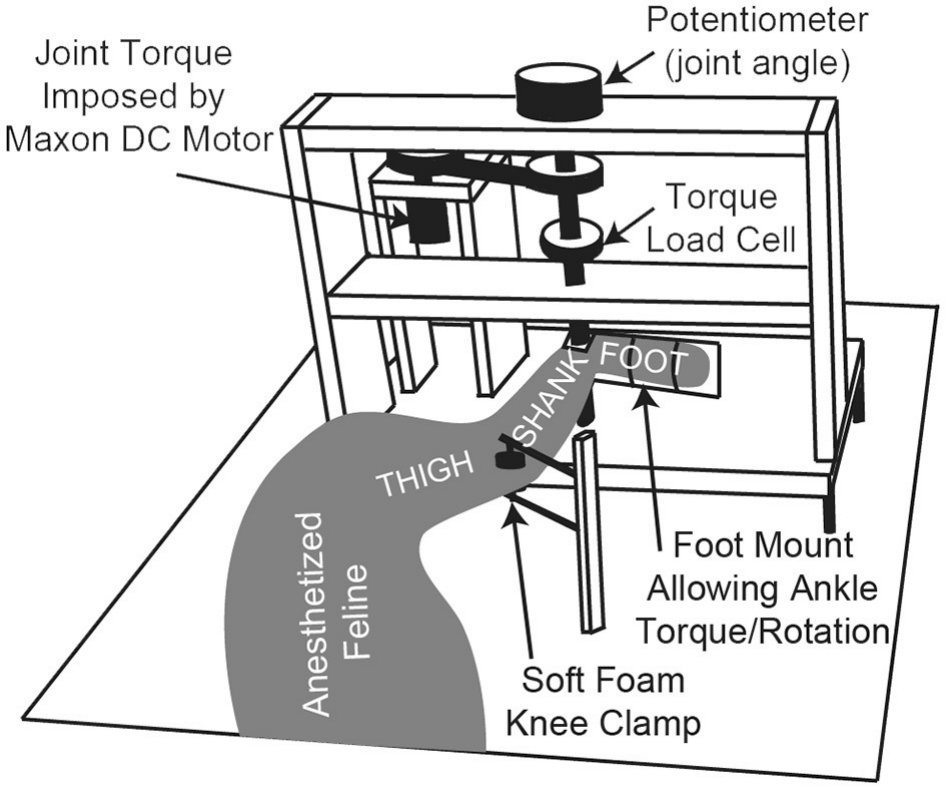
**A schematic diagram of the ankle torque control system and angular measurement apparatus**. The animal's foot is secured to the rotating foot mounting plate, ensuring that the ankle joint is concentric with the plate's center of rotation. The system electronics and an Arduino microcontroller (not shown) are mounted in the back of the setup near the DC motor.

The anesthetized animal was placed on its right side on a heated foam pad and secured, schematically shown in Figure 1. There were no joint loading effects due to gravity because the animal was horizontal. The only motion-resistive joint torque was provided by the ankle torque and angle control system, shown in Figure 1. The left foot was secured via plastic zip ties to the rotating foot mounting plate so that the center of rotation of the ankle was concentric with the center of rotation of the foot mounting plate. The left knee was secured in a soft foam clamp to prevent knee rotation, and the animal was positioned so that the left knee was at 90 deg.

### Stimulation and recording setup

Monophasic constant-voltage (−5 V) stimulation was delivered via USEA electrodes using a custom-built, multi-channel stimulator, using stimulus pulse durations between 0.2 and 1024 μs with 0.2-μs resolution (Hiatt et al., 2010). The ground return path for stimulation was the metal shell of the chronic implant connector situated subcutaneously near the center of the thigh. Stimulus pulse durations were controlled via custom MATLAB routines (Mathworks, Natick, USA). Ankle joint torque was measured by an inline loadcell (TQ201-50, Omegadyne, Stamford, USA), and ankle angular position was measured by a high-precision potentiometer. For initial twitch-force recruitment mapping and axonal activation overlap determination (described below), the torque loadcell output was sampled at 10 kHz using a Cerebus multi-channel data acquisition system (Blackrock Microsystems, Salt Lake City, USA).

For closed-loop tests, the joint-angle potentiometer output was sampled at 2 kHz using custom MATLAB software, via an NI PCI-6040E data acquisition card (National Instruments, Austin, USA). All presented data were postprocessed in MATLAB using a forward and backward 4th-order Butterworth low-pass filter with a 50 Hz cutoff frequency.

### USEA calibration

For the initial USEA calibration (Wilder et al., 2009; Dowden et al., 2012), the foot mounting plate was locked to ensure that all evoked muscle forces were isometric. The ankle angle was set at approximately 90 deg., which sets the fast-twitch ankle-plantar flexion calf muscles near a maximal force-generation length. The zero reference angle for the ankle is the tibia and increasing position is in the plantar-flexion direction. Twitch-torque recruitment maps were generated for all electrodes that evoked a peak torque greater than 0.01 Nm in response to a single 512-μs stimulus. The pair-wise level of axonal activation overlap was then measured for all electrodes whose stimulation activated fast-twitch ankle plantar-flexion calf muscles (McDonnall et al., 2004; Dowden et al., 2012). Six electrodes activating fast-twitch ankle plantar-flexion muscles with the least amount of axonal overlap were chosen for further experiments.

The control strategy used in this experiment required knowledge of the time from stimulation via each USEA electrode to the peak of the evoked twitch-torque response (Frankel et al., 2011), and this time-to-peak-response metric (*T*_*pr*_) was measured for each of the six electrodes with the ankle joint-angle fixed at 90 deg., following the procedures discussed in the prior study. Because the closed-loop experiments involved non-isometric contractions, and because *T*_*pr*_ changes as the muscle length changes (Close and Luff, 1974), the relationship between *T*_*pr*_ and ankle joint-angle was determined in early studies by measuring the *T*_*pr*_ for fast-twitch plantar-flexion muscle fibers when the ankle was held fixed at different joint angles. Although the relationship found in (Close and Luff, 1974) was non-linear, our closed-loop controller used a linear equation that adjusted the expected *T*_*pr*_ for each stimulation from (*T*_*pr*_ +5) ms when the joint-angle was 20 deg. to (*T*_*pr*_ −5) ms when the joint-angle was 160 deg.

### Controller design

For closed-loop experiments, the foot mounting plate was released and allowed to rotate, and a plantar-flexion resistive torque was generated by a geared DC motor (A-max 26, Maxon Precision Motors, Fall River, USA) controlled by a microcontroller (Arduino Uno, Smart Projects, Italy), schematically shown in Figure 1. Asynchronous stimulation of the six electrodes at a composite 60 Hz stimulation frequency was used for all closed-loop tests (10 Hz per electrode), and the stimulation phasing was set and fixed such that the predicted *T*_*pr*_ due to stimulation via each electrode, measured at 90 deg. ankle angle, would be 1/60 s after the *T*_*pr*_ due to the stimulation via the prior electrode, thus equally spacing the peak responses across each period of six-electrode stimulation (Frankel et al., 2011).

The stimulus pulse duration that was just below the torque producing threshold, along with the lowest stimulus pulse duration that evoked the maximal twitch-torque, were determined from twitch-torque recruitment curves measured for each electrode. These were set as the minimum and maximum allowable stimulus pulse durations for each electrode, creating a bounded input system (Frankel et al., 2011). Twitch-torque recruitment curves are sigmoidal in shape and the maximal twitch-torque was determined as the point at which continued increases in stimulus pulse duration evoked no substantial increase in torque, denoted as the torque plateau (Dowden et al., 2012). The slope of the twitch-torque recruitment curve between 20 and 80% of maximal twitch-torque (the “linear” range) was also determined for each electrode, and this slope was used by the controller as a per-electrode gain Equation (2) to normalize for different recruitment curve slopes amongst the six electrodes in use (Frankel et al., 2011).

**Figure 2:**
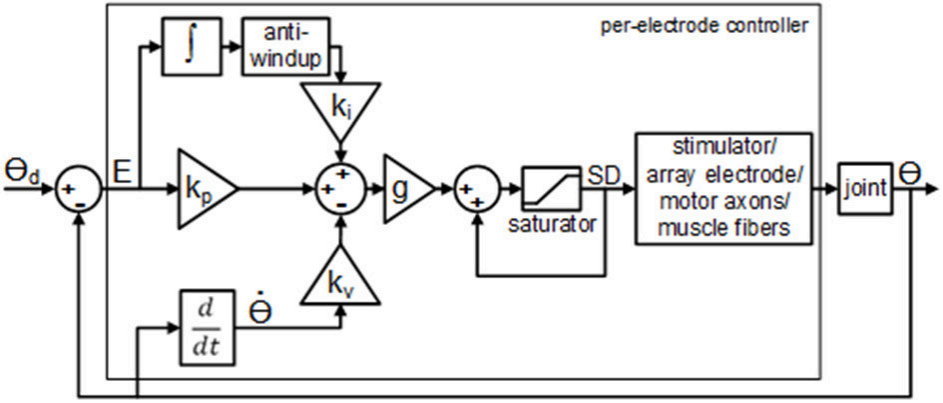
**Block diagram of the per-electrode PIV controller with integrator anti-windup**. The proportional error gain, k_p_, the joint velocity gain; k_v_, and the integral of the error gain; k_i_, are all constant linear terms. The per-electrode stimulus duration, *SD*, was bound between a pre-threshold stimulus level and a maximum allowable stimulus level.

The real-time closed-loop aIFMS control strategy and system was previously created for evoking desired isometric forces (Frankel et al., 2011), and was extended and adapted for these experiments to a dynamic motion PIV controller, schematically shown in Figure 2. The overall control law used for these experiments was

(1) $$\begin{array}{l} {SD}_{e,j+1}={SD}_{e,j}\;+\;g_{e}\;\cdot \;\left(\begin{array}{l} k_{p}\;\cdot \;E_{e,j}\\ +k_{i}\;\cdot \;\overset{n}{\underset{l=0}{\sum }}E_{e,j-l}\;\cdot \;△t\\ -k_{v}\;\cdot \;\frac{\theta_{e,j}-\theta_{e,j-1}}{△t} \end{array}\right) \end{array}$$

where *SD*_*e, j*_ is the stimulus pulse duration for the *e*-th electrode during the *j*-th stimulation cycle, *k*_*p*_ is the proportional gain, *k*_*i*_ is the integral gain, *k*_*v*_ is the velocity gain, *E*_*e, j*_ is the joint-angle error determined by Equation (3), θ_*e, j*_ is the joint-angle position, *g*_*e*_ is an additional gain factor based on experimentally determined recruitment-curve slopes for each electrode, determined by Equation (2), and Δ*t* is the time step between per-electrode stimulations (100 ms). The limit of summation, *n*, was initially set to *j*−1, which allows for complete integration across all error samples. Adjustments to this limit, which provided closed-loop response improvements, will be described in the following sections.

Similarly to what was done for force-feedback control (Frankel et al., 2011), the additional per-electrode gain was determined as

(2) $$\begin{array}{l} g_{e}=\log_{10}\left(1/s_{e}\right), \end{array}$$

where *s*_*e*_ is the slope of the per-electrode twitch-torque recruitment curve over 20–80% of maximal twitch-torque. After *g*_*e*_ was determined for all electrodes, the values were normalized to the largest value, giving *g*_*e*_ values of 0.5–1.0 for this experiment. The per-electrode error was determined as

(3) $$\begin{array}{l} E_{e,j}=\left(\overset{t=T_{{pr}_{e,j}}+w}{\underset{t=T_{{pr}_{e,j}}-w}{\sum }}\left[\theta_{d}\left(t\right)-\theta_{m}\left(t\right)\right]\right)/\left(2w+1\right) \end{array}$$

where θ_*d*_ is the desired joint-angle position, θ_*m*_ is the measured joint-angle position, *t* is a discrete time variable (sample), 2*w* + 1 is the length of the sampling window over which the error function is evaluated, where *w* was set to 5 ms of samples in all experiments, and *T*_*pr*(*e, j*)_ is the predicted time of peak twitch-torque for the *e*-th electrode during the *j*-th cycle of stimulation (expressed in samples, approximately 30 ms for electrically-activated fast-twitch muscle fibers). The joint-angle position in Equation (1), θ_*e, j*_, was determined as the mean of θ_*m*_, determined over the same sampling window used in Equation (3).

### Closed-loop studies

To tune the PIV controller, i.e., to determine the controller gains *k*_*p*_, *k*_*v*_, and *k*_*i*_, a 30-deg. step in ankle angle was used as the desired joint-angle trajectory with an oppositional torque of 0.4 Nm and a 25-deg. ankle joint-angle starting position. The oppositional torque of 0.4 Nm was chosen because this was approximately what would be required by the ankle joint of this animal to overcome gravity and generate stance. The 25-deg. starting position was the position at which the torque generated by the stiffness of the ankle muscle tendons matched the oppositional torque.

First, using only proportional control, the proportional gain was slowly increased to drive the system from overdamped to highly underdamped kinetics with a fast, stable, transient response. Second, using proportional-plus-velocity control (PV), the velocity gain was slowly increased until the system was near critically damped, which reduced the transient overshoot and decreased the settling time. The velocity term in Equation (1) is made negative because the damping is created by opposing the speed of the motion; thus, increased velocity results in decreased stimulus intensity. Finally, using proportional-plus-velocity-plus-integral control (PIV), the integrator gain was slowly increased until the overshoot reached 10%.

In these experiments, the integrator was used to drive the steady-state error toward zero for desired step motions. Although the integrator works well at reducing steady state error, it can also cause large overshoot due to integrator windup. Integrator windup often occurs when there is a large and rapid change in a desired response, e.g., during a stepped change in the desired response, and the integral of the error accumulates substantially during the rising phase of the closed-loop system response, often causing large overshoot that is not released until the integrator is unwound by error in the opposite direction. In our system, additional integrator windup occurred because of the inherent delay in the system due to the controller looking one full cycle backward in time, and because the initial stimulus intensity was pre-threshold, requiring time for the controller to increase the stimulus intensity high enough to overcome the oppositional loading torque.

To reduce the integrator windup, the integrator was made leaky, in effect turning the integrator into a lag compensator (Nise, 2004). This was done by dropping out early acquired error values from the numerical integration, i.e., only the *N* most recently acquired error values were summed; *n* = *N*−1 in Equation (1). The value of *N* was empirically determined to be eight error samples (800 ms) because this created a critically-damped response. Also, because the reduction in joint position was based solely on relaxation of muscle fibers against the joint loading torque, the integrator tended to cause an oscillatory response near a stationary desired position, which was reduced by removing the integrator for error values less than zero, i.e., when the evoked response was greater than the desired response. These improvements were implemented by modifying the control law of Equation (1) so that the limit of summation, *n*, was set to 7, and by setting *k*_*i*_ = 0 when *E*_*e, j*_ < 0.

Various step sizes from 15 to 75 deg. were then tested along with various loading torques ranging from 0.2 to 0.8 Nm, using the tuned PIV closed-loop controller gains and the adapted control law of Equation (1) as described above. For these desired step responses, the closed-loop system was experimentally evaluated for the overall evoked joint position percent overshoot, rise time, time-to-peak, settling time, and steady-state error. Percent overshoot (*%OS*) was measured as the percent difference between the peak evoked position and the mean evoked position during the last half-second of stimulation (steady-state position). Rise time (*T*_*r*_) was measured as the time from step onset to when the system reached 90% of the steady-state position. Time-to-peak (*T*_*p*_) was measured as the time from the step onset to when the system reached the peak evoked position. Settling time (*T*_*s*_) was measured as the time from the step onset to when the system settled to within ±2% of the steady-state position. Steady-state error (*SSE*) was measured as the difference between the steady-state position and the desired step position.

The controller was additionally tested for time-varying joint-angle trajectories. For desired ramp-up, hold, then ramp-down joint trajectories ranging from 2 to 64 deg./s, the closed-loop system was experimentally evaluated for time delay and amplitude error. After each trial, both the evoked ramp-up phase and the evoked ramp-down phase were shifted backward in time until the sum of the squared per-sample difference between the time-shifted evoked response and the desired joint-angle trajectory was a minimum. This time shift was evaluated as a time delay metric (*T*_*d*_) of the closed-loop system. Amplitude error (*E*_*a*_) was also used as a performance metric and was determined as the per-sample difference between the time-shifted evoked response and the desired joint position (Frankel et al., 2011). For desired sinusoidal joint trajectories, the closed-loop system was experimentally evaluated for time delay (*T*_*d*_), phase delay (Φ_*d*_), and peak-to-peak response amplitude for 0.05–0.4 Hz trajectories, which are within the bounds of normal periodic human lower limb movements that occur at speeds up to a maximum of 2.0 Hz (Murray, 1976). This analysis was done by estimating the components of the evoked response waveform that was correlated with the desired trajectory using a least-squares method.

For all closed-loop studies, multiple trials were performed for each of the scenarios described above and representative data are presented in the following results section.

## Results

### PIV gain tuning

**Figure 3:**
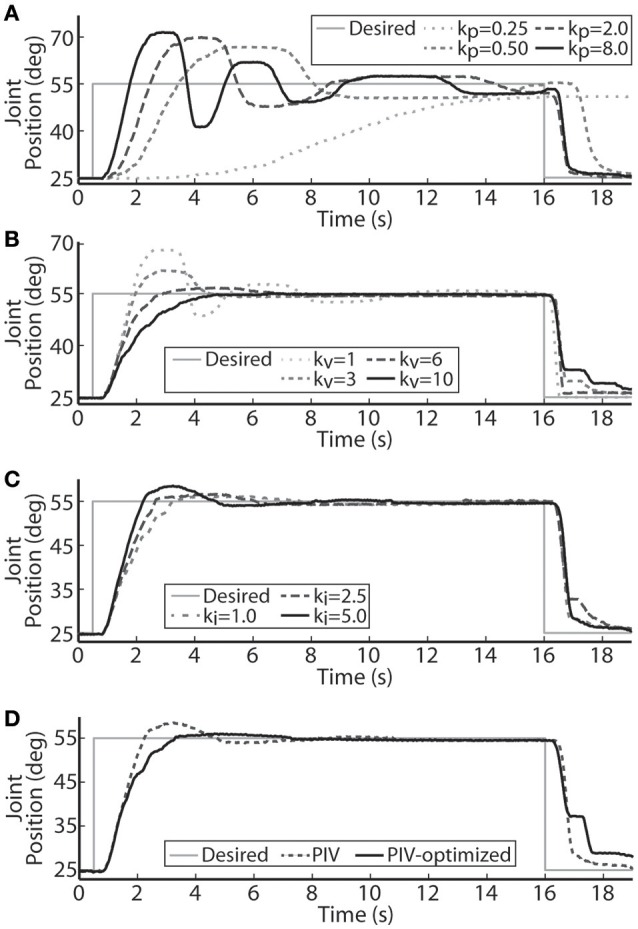
**Representative joint position responses to proportional-only control (A), proportional-plus-velocity (PV) control (B), proportional-plus-velocity-plus-integral (PIV) control (C), and tuned PIV-control with included integrator improvements (D). (A)** Changes in the closed-loop system response are shown for increases in the proportional gain, k_p_ (μs/deg.). **(B)** Changes in the closed-loop system response are shown for increases in the velocity gain, k_v_ [μs/(deg./s)], with k_p_ = 8.0 μs/deg. **(C)** Changes in the closed-loop system response are shown for increases in the integrator gain, k_i_ [μs/(deg.· s)], with k_p_ = 8.0 μs/deg. and k_v_ = 10.0 μs/(deg./s). **(D)** Controller control gains fixed at k_p_ = 8.0 μs/deg., k_v_ = 10.0 μs/(deg./s), and k_i_ = 5.0 μs/(deg.·s).

The performance of the joint-angle feedback control system was first studied using proportional-only control. Representative proportional control (P control) closed-loop system responses for several values of the proportional gain, *k*_*p*_, are displayed in Figure 3A. Using a loading torque of 0.4 Nm, *k*_*p*_ was tuned until the system showed a highly underdamped response with *T*_*r*_ = 1.18 s, *T*_*p*_ = 2.42 s, and *%OS* = 55.23%. Because the response never fully reached a steady-state, the steady-state position was estimated to calculate rise time. This steady-state estimate was determined as the mean position during the window of time between the last positive and negative peak.

Velocity control was then added to the controller (PV control) to reduce the transient overshoot and oscillatory behavior caused by the large proportional gain. Representative PV closed-loop system responses for various values of the velocity gain, *k*_*v*_, are shown in Figure 3B, with *k*_*p*_ held constant at 8.0 μs/deg. The velocity gain was tuned until the system was nearly critically damped, yielding a response with *T*_*r*_ = 2.95 s, *T*_*p*_ = 6.13 s, *%OS* = 0.33%, and *SSE* = 0.63% or 0.19 deg.

Integral control was then added to the controller (PIV control) to ensure that the steady-state error stays near zero. Representative PIV closed-loop system responses for increasing values of the integrator gain, *k*_*i*_, are presented in Figure 3C, with *k*_*p*_ held constant at 8.0 μs/deg. and *k*_*v*_ held constant at 10 μs/(deg./s). The integrator gain was tuned to produce a faster system response, *T*_*r*_ = 1.55 s, with transient overshoot of 11.38%. Integral control is not commonly used to speed up the transient response, though it had that effect in these experiments due to system delays leading to large integrator windup. To reduce settling time, the integrator was turned off when the error was less than zero (descending motion). This resulted in the settling time decreasing from 6.98 s to 6.02 s without affecting any other response metrics. The integrator was then made leaky to deal with integrator windup, i.e., only the 8 most recently acquired error values were numerically integrated, and only when the error was greater than zero.

A representative tuned PIV closed-loop system response is demonstrated in Figure 3D with the following response characteristics: *T*_*r*_ = 2.28 s, *T*_*p*_ = 4.22 s, *T*_*s*_ = 4.53 s, *%OS* = 2.39%, and <1% *SSE*, using the following controller gains: *k*_*p*_ = 8.0 μs/deg., *k*_*v*_ = 10.0 μs/(deg./s), *k*_*i*_ = 5.0 μs/(deg.·s). Table 1 summarizes the response characteristics for the PIV gain tuning.

**Table 1:** **Representative step response output characteristics for the different controllers tested**.

**Controller**	**T*_r_* (s)**	**T*_p_* (s)**	**T*_s_* (s)**	**%OS**	**SSE (%)**
P-control	1.18	2.42	N/A	55.23	N/A
PV-control	2.95	6.13	N/A	0.33	0.63
PIV-control	1.45	2.73	6.98	11.38	0.92
PIV-optimized	2.28	4.22	4.53	2.39	0.77

### Controller robustness

Using the tuned PIV controller gains, the robustness of the controlled response to various step sizes and loading torques was tested along with the ability of the controller to reject disturbance torques. In addition, the reliability of the controlled response over experimental sessions was tested using the same six electrodes and controller gains.

**Figure 4:**
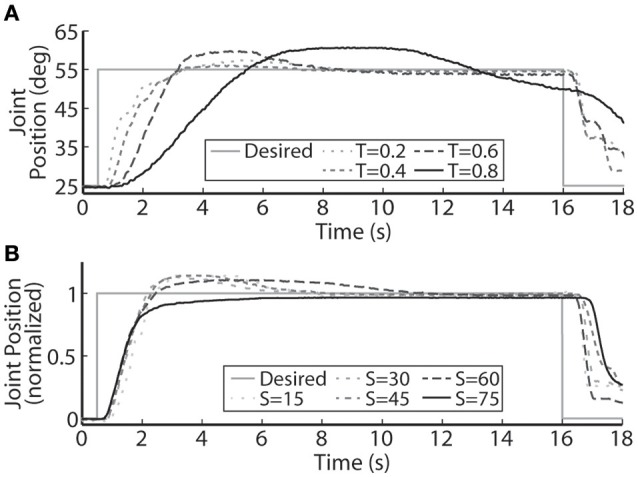
**A representative PIV controlled response to (A) increasing oppositional loading torques (T, measured in Nm), and (B) various sizes of the desired step in joint position (S, measured in deg.). (A)** At the highest loading tested, 0.8 Nm, the system was unable to settle to a steady-state value because the activated muscle fibers began fatiguing and the maximal stimulation level was reached.

The PIV controlled response for a 30-deg. desired step with increasing loading torques is displayed in Figure 4A, and summarized in Table 2. As the loading torque increased to 0.6 Nm, the controlled response took longer, *T*_*r*_ = 2.31 s, *T*_*p*_ = 4.68 s, *T*_*s*_ = 8.41 s, and had more overshoot, *%OS* = 15.6%. The PIV controlled response for several desired step sizes against a loading torque of 0.4 Nm, and with a starting position of 25 deg., is presented in Figure 4B and Table 3. The system response to a desired step size of 15 deg., 30 deg., and 45 deg. was similar, while for the 60-deg. desired step, the system response had similar rise characteristics, although slower in settling. Although close, the closed-loop system was unable to fully reach or cross a desired 75-deg. desired step (25–100 deg.), even when the controller increased the stimulation amplitude to the maximum for all six electrodes.

**Table 2:** **Representative step response output characteristics for the optimized controller with varying loads**.

**Load (Nm)**	**T*_r_* (s)**	**T*_p_* (s)**	**T*_s_* (s)**	**%OS**	**SSE (%)**
0.2	2.08	4.91	7.32	7.76	0.70
0.4	2.02	4.44	6.10	3.36	1.33
0.6	2.31	4.68	8.41	15.65	3.23
0.8	4.28	8.93	N/A	18.43	N/A

**Table 3:** **Representative step response output characteristics for the optimized controller with varying step size**.

**Step-size (deg)**	**T*_r_* (s)**	**T*_p_* (s)**	**T*_s_* (s)**	**%OS**	**SSE (%)**
15	1.88	3.59	6.42	11.91	0.73
30	1.67	2.57	6.38	11.07	1.30
45	1.76	3.15	6.50	12.19	0.44
60	1.78	4.53	10.74	8.52	1.80
75	2.03	N/A	6.48	N/A	3.63

**Figure 5:**
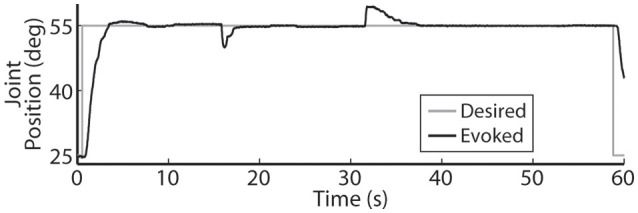
**The PIV controller was successful in responding to disturbance torques that were added at ***t*** = 16 s and then subsequently removed at ***t*** = 31 s**.

A representative closed-loop controlled response to an applied disturbance torque is demonstrated in Figure 5. The system response was initially allowed to settle against a loading torque of 0.4 Nm and then the loading torque was increased to 0.6 Nm at *t* = 16 s. The increased load was maintained for 15 s and then the loading torque was decreased from 0.6 to 0.4 Nm. When the disturbance load was initially applied, the system settled in 1.88 s with near-zero overshoot, and when the disturbance load was removed, the system settled in 5.56 s, also with near-zero overshoot.

**Figure 6:**
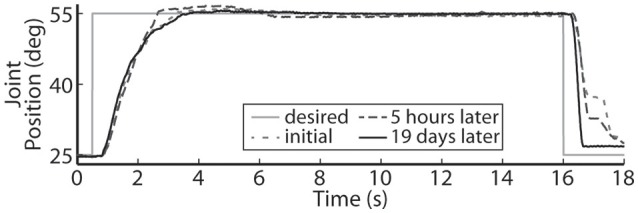
**The representative closed-loop system response remained consistent over time during a single experiment and over experimental days**.

**Figure 7:**
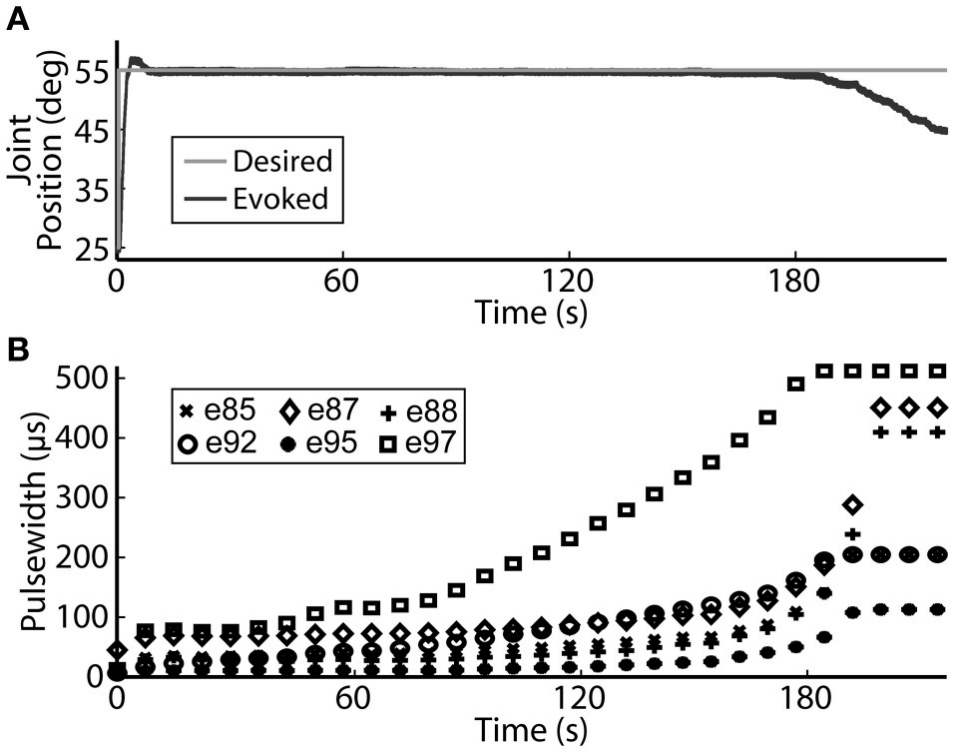
**The PIV closed-loop controller successfully modulated per-electrode stimulation intensities (pulsewidth) to compensate for the effects of fatigue. (A)** The activated muscle fibers began to fatigue and the controller compensated by increasing the stimulation intensity delivered via all six electrodes **(B)** to hold the desired joint position. Eventually, the activated muscle fibers were unable to evoke enough joint torque to hold the desired joint position against the loading torque, even with maximal stimulation.

Using the same six electrodes and PIV controller gains, representative closed-loop controlled responses over time are shown in Figure 6. When tested with a 30-deg. step in joint position against a 0.4-Nm load, the initial response, the response 5 hours later during the same experiment, and the response during a subsequent experiment 19 days later were similar: *T*_*r*_ = 2.07 ± 0.19 s, *T*_*p*_ = 4.13 ± 0.16 s, *T*_*s*_ = 6.68 ± 0.45 s, *%OS* = 3.65 ± 1.55%. The PIV controller was tested to the limit where activated muscle fibers showed strong fatigue, as presented in Figure 7.

### Time-varying trajectories

**Figure 8:**
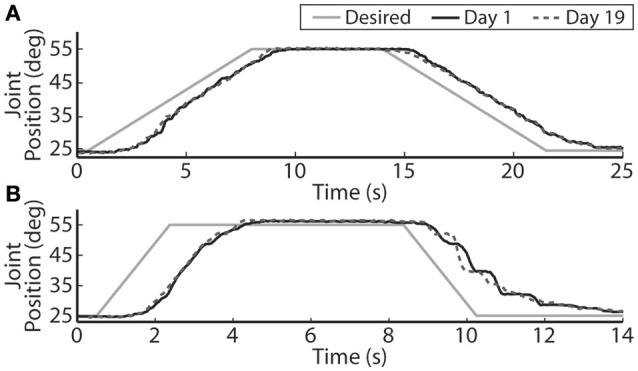
**The PIV closed-loop controller evoked time-varying ramps in joint position**. Representative results are shown for a 4-deg./s ramp in joint-angle **(A)**, and a 16-deg./s ramp in joint-angle **(B)**, for the initial day trial and the trial 19 days later using the same electrodes and controller gains.

The closed-loop PIV controller was able to evoke ramped joint-angle trajectories with slopes ranging from 2 to 64 deg./s against a 0.4-Nm loading torque, as presented in Table 4. The controlled response was smooth and accurate for slower ramp speeds, such as the 4-deg./s trajectory shown in Figure 8A, with amplitude error and time delay of *E*_*a*_ = 0.21 ± 1.02 deg., *T*_*d*_ = 1.25 s for the ramp-up phase and *E*_*a*_ = −0.24 ± 0.98 deg., *T*_*d*_ = 1.45 s for the ramp-down phase. The time delay remained at approximately 1.3 s when the speed of the desired ramp trajectories increased. However, the error of the controlled response increased and the smoothness of the evoked motion decreased, as shown in Table 4 and Figure 8B. When the desired ramp speed reached 16 deg./s, the profile of the evoked response resembled that of earlier desired steps (Figure 3D), with a rapid rise and slower settling phase for the ramp-up and a more stepped response for the ramp-down.

**Table 4:** **Representative controller-evoked response characteristics for desired ramped joint-angle trajectories (Day 1)**.

**Ramp slope (deg/s)**	**Error amplitude (deg, μ ± SD)**	**Time delay (s)**
+2	0.46 ± 0.81	0.74
+4	0.21 ± 1.02	1.25
+8	0.44 ± 1.25	1.25
+16	0.10 ± 1.28	1.30
+32	0.11 ± 2.33	1.31
+64	−0.12 ± 4.27	1.27
−2	−0.85 ± 0.49	1.07
−4	−0.24 ± 0.98	1.45
−8	−0.18 ± 2.07	1.44
−16	−0.59 ± 2.45	1.39
−32	−1.01 ± 2.53	1.29
−64	−0.24 ± 6.06	1.35

**Figure 9:**
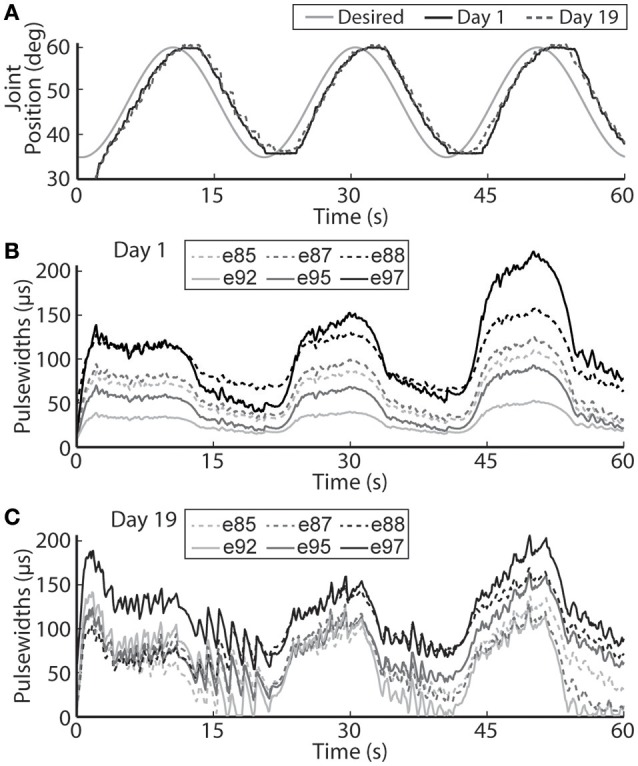
**The PIV closed-loop controller-evoked time-varying periodic joint trajectory**. Representative results are shown for a 0.05 Hz joint-angle trajectory for the initial day trial and the trial 19 days later using the same controller gains **(A)**, along with the required stimulation pulsewidths for each utilized electrode during the initial trial **(B)** and the trial 19 days later **(C)**.

**Figure 10:**
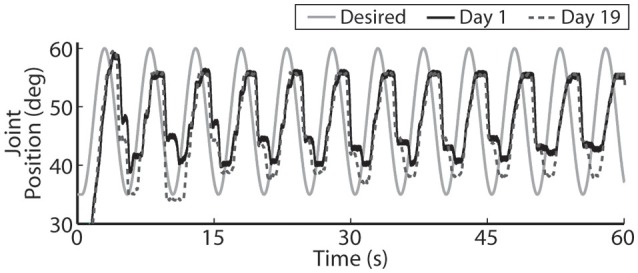
**For faster moving periodic trajectories, such as the 0.2 Hz sinusoid shown, the controller had difficulty evoking the desired amplitude and sinusoidal profile, and the descending motion became more stepped**. Representative results are show for both the initial day trial and the trial 19 days later using the same controller gains.

The closed-loop PIV controller was also able to evoke sinusoidal joint-angle trajectories with frequencies ranging from 0.05 to 0.4 Hz against a 0.4-Nm loading torque, as displayed in Table 5. The controller accurately evoked a 0.05 Hz, 35–60 deg. sinusoidal joint-angle trajectory with time delay of *T*_*d*_ = 1.46 s and phase delay of Φ_*d*_ = 26.3 deg., as shown in Figure 9A, using stimulation pulsewidths shown in Figures 9B,C. The desired sinusoidal trajectories started 10 deg. from the starting position because during stimulation the activated muscles and associated tendons stiffen, making it difficult for the system to return to the starting position when stimulation was turned off. This can be seen by the flat valleys of the evoked response in Figure 9A. The controller had difficulty evoking the full magnitude of the sinusoidal joint motion when the desired frequency increased, as shown in Figure 10 and Table 5, and although the time delays decreased, the associated phase delay increased.

**Table 5:** **Representative controller-evoked response characteristics for desired sinusoidal joint-angle trajectories (Day 1)**.

**Desired frequency (Hz)**	**Time delay (s)**	**Phase delay (deg)**	**Evoked peak-peak magnitude (deg)**
0.05	1.46	26.4	25.21
0.1	1.22	43.9	22.09
0.2	0.99	69.4	15.62
0.4	0.79	113.8	8.78

Using the same six electrodes and PIV controller gains, the closed-loop controlled response for the time-varying trajectories remained consistent over time, as shown in Figures 8–10, even though the required pulsewidth profiles for each electrode were substantially different, as shown for the 0.05 Hz sinusoidal trajectory in Figure 9C.

## Discussion

The results presented in this paper demonstrate that tracking of desired step, ramp, and sinusoidal joint-angle trajectories can be successfully achieved with real-time joint-angle feedback control of aIFMS. This demonstration is important because although prior experiments evoked precise time-varying isometric muscle force trajectories (Frankel et al., 2011), many real-world motions involve the generation of dynamic non-isometric muscle forces that are complicated by non-linear dependencies of muscle force on muscle length and velocity. Because of these complexities, proportional-only control was not sufficient and it was necessary to adapt the proportional-only controller (Frankel et al., 2011) to the proportional-integral-velocity controller with integrator anti-windup used in this study. The set of desired trajectories used to evaluate the controller have real-world relevance because lower-limb neuroprostheses will need to robustly evoke controlled stances (ramped and stepped motion) and gait (periodic motion). It is important to note that the subtle additions and changes to a standard PID controller, including using a velocity term instead of error derivative and using a leaky integrator with anti-windup only when the error was positive, made substantial improvements to the system response that were not initially predicted (Figure 3D).

It is also very important to note that on the second day of experiments, 19 days after the initial tests, the controlled results for all tested trajectories remained consistent without having to do initial USEA characterization or change the controller gains (Figures 6, 8–10). Although the evoked 0.05 Hz sinusoidal response was similar on day 1 and day 19, the required pulsewidth profiles were substantially different for each of the utilized electrodes, which can be notably seen for electrodes e88 and e97 in Figures 9B,C. This demonstrates the controller's ability to evoke desired trajectories even though there may be subtle changes to the interface between the electrode and nerve, including tip movement and axonal death and growth. It is critically important for a clinically-viable FNS-based neuroprosthesis that the closed-loop controlled response be consistent over time, be robust to changes in loading torque and/or desired motion, be resistive to external disturbances and muscle fatigue, and be able to handle other unknown changing physiological conditions. Stable controller gains would reduce the need for daily controller tuning or rule-based control for specific motions.

With increased loading torque, the system required more muscle fibers to be activated to evoke enough joint torque to initiate motion. In terms of the PIV controller, this lead to more integrator windup before enough stimulation was delivered to generate motion (Figure 4A), which can lead to large transient overshoot (Figure 3C). To reduce the overshoot, the integrator was made leaky, i.e., early acquired error values were dropped from the numerical error integration over time, in effect turning the integrator into a lag compensator (Figure 3D). This successfully reduced the transient overshoot while allowing early integration windup in the controller to recruit the necessary muscle fibers. The leaky integrator was also useful for evoking smooth time-varying joint trajectories (Figures 8A, 9A). There will always be some delay in the closed-loop response because the controller is looking backwards in time and this inherent delay would likely lead to large integrator windup and less smooth evoked motion.

The controller was also able to handle externally applied disturbance torques (Figure 5). When the disturbance torque was added, the controller was able to rapidly drive the system back to steady-state with near-zero overshoot. However, when the resistance torque was removed, the controlled response took longer to return to steady-state. This increased settling time was due to the lack of integrator control during descending motion.

Electrically stimulating motor nerves to evoke muscle forces can be limited by rapid muscle fatigue due to high stimulation frequencies required and the inverse recruitment nature of FNS, where fast-fatiguing muscle fibers are preferentially activated before slow-fatiguing fibers. aIFMS provides fatigue resistance by asynchronously activating small subsets of motor fibers within a targeted muscle at a low per-subset frequency (McDonnall et al., 2004; Normann et al., 2012), though eventually all activated muscle fibers will fatigue. The PIV-controller was successful in compensating for the fatigue of activated muscle fibers by rapidly increasing stimulation to recruit more muscle fibers and hold a desired joint position (Figure 7). Eventually, all activated muscle fibers showed strong fatigue against the 0.4 Nm load and the controller was unable to recruit more muscle fibers after reaching maximal stimulation. This could create problems for continuous high-force generation during experimental sessions, and safety measures should be created to recognize increasing error even with increasing stimulation.

By using the velocity of the evoked joint-angle trajectory to create damping, as opposed to the derivative of the error which is often used (Nise, 2004), the controller avoided a large error derivative that occurs at the discontinuities in desired position for step trajectories, which is not often an issue for fast responding systems but can cause problems for slower responding systems such as the 10 Hz per-electrode controller. Our PIV strategy resulted in smoother evoked step-up motion. However, the large velocity gain did cause a more stepped downward motion, which was seen in many of the results. When there was a large negative error, e.g., during a step-down phase, the controller rapidly decreased the intensity of stimulation delivered via the USEA electrodes, attempting to allow rapid muscle fiber relaxation and descending motion. In response to this, because of the large velocity gain and the fact that the integrator is turned off during descending motion, the controller attempted to dampen and slow this rapid descent by activating more muscle fibers, causing the downward motion to manifest the stepped descending trajectory. This may be compensated for in future work by the addition of antagonist muscle control.

One of the major limitations of current clinical FNS-based prostheses is the inability to evoke graded muscle force due to their on/off nature (Bhadra and Peckham, 1997). This makes it difficult for a lower-limb prosthesis to smoothly control a sit-to-stand motion or the eccentric stand-to-sit motion, something a paralyzed individual will require in a lower-limb neuroprosthesis. Toward this goal, our real-time, PIV-controlled aIFMS system was successful in evoking smooth slow-moving, time-varying joint motions with low error and short time delay (Figures 8A, 9A). However, the controller struggled to evoke more rapidly changing desired positions (Figures 8B, 10). In earlier studies using proportional-only control of isometric forces (Frankel et al., 2011), we were able to evoke faster time-varying forces up to 2 Hz with shorter time delays, though only after increasing and tuning the proportional controller gain. This study was designed to evaluate the PIV controller-evoked responses using only one set of tuned controller gains. It may be possible to evoke higher frequency sinusoidal trajectories with more accuracy and less delay with different PIV controller gains, though this would likely require substantial time to tune for each desired frequency. This is an area that will need to be addressed with future controller designs because normal human movement can often have components as fast as 2 Hz (Murray, 1976) with reaction time delays of ~200 ms (Welford, 1980), and although prior research using muscle spindle activity as an estimator for joint position and PI feedback control of the feline ankle angle found similar difficulties tracking higher frequency joint trajectories (Yoshida and Horch, 1996), other researchers have been able to accurately generate up to 0.8 Hz FNS-controlled feline walking (Mazurek et al., 2012).

The controller-evoked results were similar across different desired step sizes (Figure 4B), though the system was unable to achieve a 25–100 deg. step although this is not outside the movement range for feline ankles (MacFadden, 2009). This may be because of electrode limitations. The USEA was not characterized until after it was chronically implanted. A small subset of approximately 10 electrodes, that selectively activated ankle plantar-flexion muscles, were viable for these experiments, as opposed to near 30 in prior studies, and these electrodes evoked smaller torques than seen in prior studies. The chronically-implanted feline used for this study was used for multiple on-going studies, and while these experiments were successful in demonstrating the utility of this control methodology, a better implant would provide more electrodes that selectively activate more motorneuron pools, likely providing stronger forces and torques, and a wider movement range.

Improvements to the closed-loop response may be achievable by using more sophisticated non-linear, adaptive, and optimal control strategies, which have been extensively studied for single-input single-output control of FNS-based motor neuroprostheses (Chizeck et al., 1988; Veltink et al., 1992; Jezernik et al., 2004; Nekoukar and Erfanian, 2010). Sliding-mode control was preliminarily tested and performed poorly due to the bang-bang nature of the controller which requires a very rapid update frequency to be effective. Additionally, adding some predictive feedforward control (Abbas and Triolo, 1997; Chang et al., 1997; Ferrarin et al., 2001; Sharma et al., 2011; Frankel et al., 2012) would likely reduce the inherent time delay of the controller, especially for ballistic motion such as during rapid changes in desired position.

It is possible that there can be changes to the twitch-torque recruitment curve over time due to effects such as electrode movement, immune response to the implant, and axonal death and growth, which might affect the per-electrode controller gain used in this control method. Although recruitment curve stability is something being investigated by multiple researchers, the stability of the desired response 5 hours after initial tests and 19 days later show that these variations may not be substantial enough to affect outcomes.

Although this study investigated real-time control of the per-electrode aIFMS stimulus intensities using a fixed stimulation frequency with fixed interelectrode phasing, it may be possible to improve the evoked responses by adding control over the stimulation frequency and interelectrode phasing. Because the controller is only able to add unidirectional force, descending motion of the joint is solely dependent on controlling the number of muscle fibers that are allowed to relax and their time-dependent dynamics. Using controlled antagonistic muscles, as is common in natural rapid movements, would likely improve the evoked descending motion (Perreault et al., 2001), though the amount of agonist/antagonist co-contraction would require additional control methodology. All of these improvements will be investigated in future experiments designed for more complex multiple muscle and multiple joint control.

The experiments were conducted 3.5 and 4 months after implantation of the USEA in the sciatic nerve and the same six electrodes were utilized during both experimental sessions. Although only single, representative trials for each scenario are presented, multiple trials were performed and outcomes were similar. Recruitment curves for all electrodes were measured for the two sessions. For the six electrodes utilized, the activation thresholds remained consistent (6–38 μs for session one and 6–44 μs for session two, 19 days later), and the maximum evoked torque remained strong (14–20 Ncm for session one and 20–25 Ncm for session two). A further experimental session was desired around 30 days from the original, but the implant was no longer viable to a degree where stimulation via these electrodes produced strong muscular output (maximum evoked torques were below 6 Ncm). These are important outcomes of these experiments that extend the successful chronic use of USEAs for stimulation and recording, while providing insights toward limitations (Branner et al., 2004; Clark et al., 2011; Frankel et al., 2011; Normann et al., 2012). The ability to have consistently stable electrodes will help minimize the need for continual controller tuning and initialization, which will be important in clinical applications, and studies on all aspects of the stability of USEA implants are ongoing.

## Conclusion

This paper demonstrated the first successful closed-loop dynamic limb position control using aIFMS delivered via a chronically implanted USEA. The approach presented in the paper, and experiments validating it, represent an important step toward creating the next generation of clinically-viable, fatigue-resistant, controlled FNS-based neuroprostheses to aid and restore lost motor function in persons with paralysis.

## Author contributions

MF was a graduate student during this research. He performed all the experiments and is the main author of this manuscript. The other four authors, VM, GC, RN, and SM, all contributed to the experimental design of this research, participated during experiments, and assisted in creation of this manuscript.

## Funding

This work was supported in part by USAMRMC W81XWH-10-1-0931, University of Utah, and Oregon State University.

### Conflict of interest statement

The authors declare that the research was conducted in the absence of any commercial or financial relationships that could be construed as a potential conflict of interest.
